# Effects of Aerobic Exercise Based upon Heart Rate at Aerobic Threshold in Obese Elderly Subjects with Type 2 Diabetes

**DOI:** 10.1155/2015/695297

**Published:** 2015-05-18

**Authors:** Gian Pietro Emerenziani, Maria Chiara Gallotta, Marco Meucci, Luigi Di Luigi, Silvia Migliaccio, Lorenzo Maria Donini, Felice Strollo, Laura Guidetti

**Affiliations:** ^1^Exercise and Sport Sciences Unit, Department of Movement, Human and Health Sciences, University of Rome “Foro Italico”, Piazza Lauro De Bosis 6, 00135 Rome, Italy; ^2^Department of Health and Exercise Sciences, Appalachian State University, Boone, USA; ^3^Endocrinology Unit, Department of Movement, Human and Health Sciences, University of Rome “Foro Italico”, , Italy Endocrinology Unit, Department of Movement, Human and Health Sciences, University of Rome “Foro Italico”, Piazza Lauro De Bosis 6, 00135 Rome, Italy; ^4^Medical Physiopathology, Food Science and Endocrinology Section, Department of Experimental Medicine, Food Science and Human Nutrition Research Unit, Sapienza University of Rome, Piazzale Aldo Moro 5, 00185 Rome, Italy; ^5^Diabetes Care Unit, St. Spirito Hospital, Lungo Tevere in Saxia 1, 00193 Rome, Italy

## Abstract

In obese diabetic subjects, a correct life style, including diet and physical activity, is part of a correct intervention protocol. Thus, the aim of this study was to evaluate the effects of aerobic training intervention, based on heart rate at aerobic gas exchange threshold (AerT_ge_), on clinical and physiological parameters in obese elderly subjects with type 2 diabetes (OT2DM). Thirty OT2DM subjects were randomly assigned to an intervention (IG) or control group (CG). The IG performed a supervised aerobic exercise training based on heart rate at AerT_ge_ whereas CG maintained their usual lifestyle. Anthropometric measures, blood analysis, peak oxygen consumption (${\dot{\text{V}}\text{O}}_{2\text{peak}}$), metabolic equivalent (MET_peak_), work rate (WR_peak_), and WR_AerTge_ were assessed at baseline and after intervention. After training, patients enrolled in the IG had significantly higher (*P* < 0.001) ${\dot{\text{V}}\text{O}}_{2\text{peak}}$, MET_peak_, WR_peak_, and WR_AerTge_ and significantly lower (*P* < 0.005) weight, BMI, %FM, and waist circumference than before intervention. Both IG and CG subjects had lower glycated haemoglobin levels after intervention period. No significant differences were found for all the other parameters between pre- and posttraining and between groups. Aerobic exercise prescription based upon HR at AerT_ge_ could be a valuable physical intervention tool to improve the fitness level and metabolic equilibrium in OT2DM patients.

## 1. Introduction

Obesity is a leading risk factor for premature mortality and chronic health hazards such as type 2 diabetes, coronary heart diseases, and hypertension. According to the World Health Organization at least 2.8 million adults die each year as a result of being overweight or obese. In addition, 44% of the diabetes burden, 23% of the ischaemic heart disease burden, and 7 to 41% of certain cancers are also attributable to overweight and obesity. The prevalence of overweight and obesity has increased to epidemic proportions in the industrialized world and it is now dramatically rising in low- and middle-income countries, particularly in urban settings. It is well known that regular physical activity (PA) provides health benefits and it is considered an essential component of primary and secondary prevention for most of metabolic-syndrome related pathologies [1, 2]. Recent experimental data suggests that subjects who increased their level of PA over time have a decreased mortality rate compared to those who were consistently unfit [3, 4].

Despite such evidence, physical inactivity remains a global health problem and its negative effects on health were well documented [5] as well as negative economic consequences [6]. The role of exercise intensity on physical training adherence is supported by several large studies [7–9]. Furthermore, Dishman and Buckworth [10] showed that activity-promotion efforts were more effective when the intensity was low rather than high. Accordingly, exercise intensity should be set in order to reach positive physiological effects, decrease the risk of injury, and increase adherence. It is well known that exercise can elicit different physiological responses in obese individuals when prescribed in absolute terms [11], since, usually, no adjustment is usually made for each individual's exercise capacity. Moreover, the use of relative terms, such as %VO_2max⁡_, has been substantially criticized [12–14], since it seems that the relative parameters alone without considering the aerobic threshold (AerT) are not enough to individualize the exercise intensity [12]. AerT is the point after which ventilation begins to increase disproportionately relative to oxygen uptake and it is considered a useful parameter for optimal aerobic exercise intensity prescription in DM2 patients [12, 15–17]. To clarify this specific terminology, Meyer et al. [14] suggested using the terms AerT_ge_ when aerobic threshold is evaluated by gas analysis.

Moreover, during the last years, AerT_ge_ has been more frequently used to prescribe exercise intensity in obese and diabetic populations [12, 14, 15, 18, 19]. Interestingly, there are two different methods to exercise at a relative AerT_ge_: the first aims at maintaining a work load, such as speed and power, corresponding to AerT, and the second one is keeping a constant HR corresponding to AerT_ge_ [20]. However, since musculoskeletal complications commonly present in diabetic patients [21] could prevent obese elderly patients from performing 30 min exercise at constant average load, it could be useful to choose an approach based on constant HR at AerT_ge_. 

The effects of 30 min exercise at constant HR on physiological parameters were previously described by Kindermann et al. [20] who showed that it is possible to maintain the optimal individual workload intensity by regulating HR during exercise. The intensity of PA has been found to be negatively related to adherence in overweight or obese subjects [22]. Moreover, DaSilva et al. [22] showed that overweight individuals choose to exercise at intensities below or around the ventilatory threshold and that this intensity results in low perceived exertion and positive affective responses. Also, frequent walking of sufficient duration performed more than 3 days a week might improve glycaemic control and lipoprotein profiles of subjects with type 2 diabetes and, also, cardiorespiratory fitness [15, 23].

To the best of our knowledge, only one study [23] evaluated the effects of heart rate intensity prescribed walking training program on cardiorespiratory fitness and glycaemic control in individuals affected by type 2 diabetes mellitus. Subjects' peak heart rates, obtained from the Balke-Ware protocol, were used to set a training intensity at the 80% HR_peak_ and only walking exercise was performed for a training period of 7 weeks.

Therefore, the aim of our study was to evaluate the effects of three months of aerobic exercise, based upon heart rate at AerT_ge_ on glycemic control, body weight, and fitness in obese elderly subjects with type 2 diabetes.

## 2. Methods

### 2.1. Participants

Thirty obese elderly subjects (age 66.8 ± 6.3 years), body mass index (BMI) of 34.6 ± 3.2 kg/m^2^, and percent fat mass (%FM) of 36.5 ± 6.8% with type 2 diabetes, were recruited by a diabetes care unit hospital. Participants provided written informed consent and protocol was approved by the Local Scientific Committee. All subjects were sedentary and they had not been previously engaged in regular physical exercise program. Diagnosis of Type 2 diabetes mellitus (T2DM) was established according to the World Health Organization criteria [24]. Subjects underwent clinical examination to rule out any contraindications to PA such as neuropathy, autonomic dysfunction, cardiovascular diseases, and high blood pressure (≥140/90 mmHg). All subjects were on oral pharmacological treatments.

### 2.2. Experimental Design

Subjects were randomly assigned to an intervention group (IG) (15 subjects, age 66.7 ± 4.9 years) or to a control group (CG) (15 subjects, age 66.9 ± 4.2 years). Pulmonary function tests and a resting electrocardiogram (ECG) recording were performed as initial screening in both groups. Prior to the first test session, participants took part in a familiarization session to become accustomed to PA tests. Anthropometric measurements, blood sampling, dietary and physiological evaluation, and a submaximal graded exercise test were performed at baseline and after 3 months in both groups. During the 3-month intervention period, IG group performed supervised aerobic exercise training while CG did not perform any organized exercise. Lifestyle and food behaviour of the subjects have not changed during the experimental procedure.

### 2.3. Body Composition

Participant's height was measured at the time of hospital referral using a stadiometer to the nearest 0.1 cm. Body composition was determined using a multifrequency bioimpedance analysis (InBody 720, Biospace Inc., Cerritos, CA, USA) [25]. All subjects were tested in the morning after 12-hour overnight fast. All body mass (BM) measurements were taken on a calibrated digital scale (InBody 720, Biospace Inc., Cerritos, CA, USA) when the subjects wore minimal clothing (i.e., underwear). Body mass index (BMI) was calculated as body mass divided by squared height (kg/m^2^).

Waist circumferences were taken at 2.5 cm above the umbilicus [26] using an inextensible metallic tape placed directly on the skin, perpendicularly to the long axis of the body, and horizontally to the floor at the end of normal expiration. Average values from two measurements were considered.

### 2.4. Blood Analysis

All subjects had 5 mL blood drawn into a vacutainer from an antecubital vein in the morning after the overnight fast. Serum obtained after clotting and centrifugation at 1500 g for 20 min was used for glucose, triglyceride, and total/HDL cholesterol determination by a Kodak Autoanalyzer. Low density lipoprotein cholesterol (LDLC) was determined using Friedwald's equation for triglycerides <400 mg/dL. Additional 2 mL blood samples were also drawn into EDTA-treated vacutainers for glycated hemoglobin (HbA1c) determination using high-performance liquid chromatography (BioRad Dia-STAT Analyzer 1).

### 2.5. Aerobic Power

${\dot{\text{V}}\text{O}}_{2\text{peak}}$ was assessed in all participants by means of a continuous, maximal graded exercise test on a cycle ergometer or on a treadmill according to the individual abilities. In particular, subjects who were able to walk safely performed a modified Balke protocol [27] on a treadmill (Run Med Excite, Technogym, Gambettola (FC), Italy), while those that were not able to walk safely performed a bike-ramp 10w protocol [28] on a cycle ergometer (Bike Med Excite, Technogym, Gambettola (FC), Italy). Heart rate (beats·min^−1^) was continuously recorded before and throughout the trial using a HR monitor (RS 400, Polar Electro, Kempele, Finland). ${\dot{\text{V}}\text{O}}_{2}$ and pulmonary ventilation ($\dot{\text{V}}\text{E}$) were measured by a semiportable gas analysis system (Fitmate Pro Cosmed, Rome, Italy) [29]. Prior to each test the Fitmate Pro underwent an automatic gas calibration cycle and the turbine flow meter was periodically calibrated using a 3 L syringe according to the manufacturer's recommendations. During the test, the highest VO_2_ attained was chosen as the peak oxygen uptake (${\dot{\text{V}}\text{O}}_{2\text{peak}}$). The individual aerobic gas exchange threshold (AerT_ge_) was determined offline for each subject by plotting the ventilatory equivalent ($\dot{\text{V}}\text{E}$/${\dot{\text{V}}\text{O}}_{2}$) as a function of ${\dot{\text{V}}\text{O}}_{2}$ to identify the point during exercise where this curve reached its lowest value [17, 30]. The level of ${\dot{\text{V}}\text{O}}_{2}$ at which we observed the lowest value of the $\dot{\text{V}}\text{E}$/${\dot{\text{V}}\text{O}}_{2}$, in the individual plot, was the individual aerobic threshold [12, 31]. Moreover, work rate (WR) and metabolic equivalent (MET) were calculated at AerT_ge_ (WR_AerTge_ and MET_AerTge_, resp.) and at maximal effort (WR_peak_ and MET_peak_, resp.).

### 2.6. Training Protocol

Subjects of the IG group performed a 3-month aerobic training (AT) twice a week based on HR corresponding to their individual AerT_ge_. All training sessions, lasting 50 min, were supervised by a PA specialist. The AT was performed on a treadmill (Run Med Excite, Technogym, Gambettola (FC), Italy) or on a cycle ergometer (Bike Med Excite, Technogym, Gambettola (FC), Italy) in accordance with the device used for the evaluation of ${\dot{\text{V}}\text{O}}_{2\text{peak}}$. Subjects' HR, corresponding to AerT_ge_ obtained from maximal exercise test, was used to set the intensity of training protocol which consisted of a 5 min warm-up, 30 min AT, and 5 min cooldown. All devices were programmed at the beginning of the training to change the external work load (inclination for the treadmill and wattage for the cycle ergometer) to maintain the subjects' HR below (warm-up and cool-down periods) or equal to (training period) the individual subject's HR at AerT_ge_. Moreover, the maximal treadmill speed was set not higher than 5 km/h to perform all training sessions as safely as possible. Subjects training on a cycle ergometer maintained the pedaling rate at 40 RPM. This pedaling rate was the same used during the incremental graded test. Stretching (5 min) exercises, involving main lower limb muscle groups, were performed after warm-up and cool-down periods.

### 2.7. Dietary and Psychological Counselling

Dietary and psychological counselling was performed by a dietitian and a psychologist, respectively, to minimize these items' variability between groups. A low-calorie diet was set at approximately 400 Kcal less than total daily energy expenditure in both IG and CG groups. Total daily energy expenditure was set according to the following equation: resting metabolic rate + physical activity level. Resting metabolic rate was estimated by the Harris benedict equation [32] while physical activity level was estimated by the international physical activity questionnaire (IPAQ) [33]

### 2.8. Statistical Analysis

The similar baseline characteristics of the two groups were verified by unpaired *t*-test at T0. Due to the between subjects variability of glycated haemoglobin at baseline (T0), a 2 × 2 mixed analysis of covariance (ANCOVA) with group (IG versus CG) as between factor, time (before versus after) as within factor, and glycated haemoglobin baseline data as covariate was performed.

For each variable, mixed ANOVA with repeated measures on time was used to detect significant effects of two main factors: group (intervention versus control) and time (before versus after). Post hoc analysis of significant differences for group factor was performed using the unpaired Student's *t*-test, while for time factor it was performed using the paired *t*-test. All statistical analyses were performed with the SPSS statistical package (Version 20.0 for Windows; SPSS Inc., Chicago, IL, USA). All tests were two-tailed, with *α* ≤ 0.05 being taken as significant.

## 3. Results

**Figure 1:**
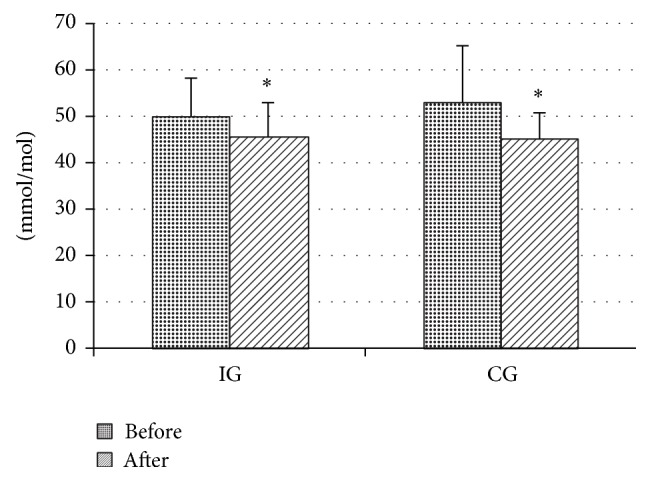
Glycated hemoglobin (HbA1c) before and after 3-month intervention period in control (CG) and intervention groups (IG).

The baseline characteristics were similar (no significant differences) between intervention and control groups for any studied variable as depicted in Table 1. Subjects in the IG reported a transitory, low muscle pain during training. A significant group × time interaction (*P* < 0.05) was found for body mass index (BMI), %FM, and abdominal circumference, indicating that the trend of these variables was different between groups after 3 months. In fact, the aerobic training based upon HR resulted in a significant decrease in BMI, %FM, and abdominal circumference, while there were not any differences in CG on anthropometric variables (Table 1). Moreover, significant group × time interaction (*P* < 0.05) was found for ${\dot{\text{V}}\text{O}}_{2\text{peak}}$, MET_peak_, WR_peak_, and WR_AerTge_, indicating that only in IG group these variables increased (*P* < 0.005) in posttraining (Table 2). A significant main effect of time was found in glycated haemoglobin that significantly lowered after 3 months in both groups (Figure 1) but no significant effects were found in total cholesterol, high density lipoprotein cholesterol (HDLC), and low density lipoprotein cholesterol (LDLC).

## 4. Discussion

The results presented herein demonstrate that individually designed exercise, at a relative AerT_ge_, improves fitness and metabolic parameters in untrained, sedentary, and obese diabetic subjects. These positive effects were controlled using baseline glycated haemoglobin as covariate.

Indeed, the first aim of this study was to determine the effects of three months of aerobic exercise training, based on heart rate at aerobic threshold, on long term glycaemic control, body composition, and exercise capacity in OT2DM. In our study, all type 2 diabetic subjects were obese and they did not practice any organized physical activity prior to this study. Moreover, subjects' fitness parameters, such as ${\dot{\text{V}}\text{O}}_{2\text{peak}}$, showed that they were very unfit. According to these observations, we chose not to use high-intensity exercise but a constant moderate intensity exercise as training method. American College of Sports Medicine PA guidelines for type 2 diabetic subjects recommend to perform low-to-moderate intensity physical activity (at 40–70% ${\dot{\text{V}}\text{O}}_{2\max}$) to achieve cardiorespiratory and metabolic improvements. Most importantly, the lower intensity activity affords a more comfortable level of exertion and enhances the likelihood of adherence, while lessening the likelihood of musculoskeletal injury and foot trauma, particularly when weight-bearing activity is recommended [34]. At present, American College of Sports Medicine suggests using three variables to monitor exercise intensity: ${\dot{\text{V}}\text{O}}_{2\max}$, HR, and rate of perceived exertion (RPE). Moreover, during the last years, the gas exchange threshold was identified as a valid tool to delineate the “training zone” for endurance training [14] and for unhealthy subjects [12].

In our exercise protocol, subjects performed 30 min aerobic exercise at an intensity corresponding to their AerT_ge_. In detail, the heart rate determined at AerT_ge_ was kept constant while the external work load, such as treadmill speed or cycle ergometer watt, decreased in accordance with HR. Kindermann et al. [20] studied the physiological responses of a constant exercise performed at a heart rate corresponding to the anaerobic threshold (4 mmol/L). In accordance with our results they showed that treadmill speed must be reduced continuously to maintain the HR constant. In our study, subjects who exercised on a cycle ergometer finished the aerobic program with a work load lower than that set at the beginning of training (decrement range 5–20 watts). In accordance, subjects who trained on a treadmill finished the aerobic program with a lower inclination than that at the beginning of training (decrement range 1–4°). The decrease in external work load was chosen to allow subjects to perform all 30 min aerobic training because we originally noticed that our obese elderly type 2 diabetic patients were not able to exercise for 30 min at a constant external work load corresponding to AerT_ge_.

It is well known that aerobic training associated with resistance exercise might improve glycemic control to an extent comparable to some oral hypoglycemic agents [35, 36]. The reduction of blood glucose in type 2 diabetic subjects could be reached by either exercising at constant moderate intensity or performing brief high-intensity exercise [15, 37]. Moreover, no data are available regarding the effects of aerobic training based upon constant heart rate at AerT_ge_ on glycemic control and physiological parameters in diabetes subjects. The reduction in glycated haemoglobin observed in IG (−9.2%) was similar to that found in other studies. For example, Belli et al. [15] found that after a 12-week supervised walking training at AerT_ge_, glycated haemoglobin decreased by 11,6%. Moreover, other two studies, Walker et al. [38] and Shenoy et al. [39], showed a decrease of glycated haemoglobin ranging from 7.6% to 9.7%, respectively. Contrary to our results, Morton et al. [23] showed no effects on glycated haemoglobin after 7 weeks of heart rate prescribed walking training. The difference between our results and those reported by Morton could be explained by the different duration of the two intervention periods. In fact, as suggested by Kilpatrick [40], glycation of haemoglobin is dependent on mean blood glucose concentrations over the 120-day lifespan of red blood cells. The lower glycated haemoglobin in CG at the end of the study than at baseline could be explained by the fact that all subjects were recruited from local health facilities and they were under pharmacological treatment since the beginning of the study. Therefore, our training protocol did not modify the positive effect on glycated haemoglobin of pharmacological treatment that was equal for both groups.

No significant differences were found in high density lipoprotein cholesterol (HDLC) and low density lipoprotein cholesterol (LDLC) levels in both groups after the training period. This was an unexpected result considering that all subjects received a low-calorie diet at baseline and that the positive effects of diet on lipid profile and weight are well known [41]. Clearly, dieting strategies provide benefits only in complying patients [42]. Indeed, a restricted diet was given at baseline to all subjects, but it was not possible to evaluate adherence to nutritional protocol throughout the study, which might lead to the hypothesis that subjects were not fully compliant to the diet intervention, as also suggested by the small, but significant decrease in body weight, fat mass, and abdominal circumference in IG group.

These results could be explained by the positive effects of physical exercise on energy expenditure. In fact, even if diet adherence was not controlled, IG performed a supervised physical training protocol leading to an energy expenditure increase during the study period.

To our knowledge the aerobic training of the subjects of our study could not be compared with any other study in the literature. In fact, to allow participants to perform a 30 min aerobic exercise we chose to maintain constant the heart rate corresponding to AerT_ge_ while the external work load decreased automatically. This method was chosen for two main reasons: firstly, untrained obese older T2DM subjects were very unfit and therefore they could not perform 30 min exercise at the constant work load corresponding to AerT_ge_; secondly, the right balance between exercise duration and intensity is a challenging task, since both factors have the potential to negatively impact adherence. PA intensity has been found to be negatively related to adherence in several studies involving overweight participants [43–45]. In fact, on average, overweight individuals choose an intensity below or around the AerT_ge_ during a 20 min bout exercise [22]. Self-paced exercise was reported to fall within the zone of fat maximal oxidation [45] and, when performed over ground, resulted in lower perceived exertion and more positive affective responses than on treadmill [7].

Our results showed that 30 min aerobic exercise based upon HR corresponding to AerT_ge_ could improve physical exercise capacity in obese T2DM subjects as demonstrated by ${\dot{\text{V}}\text{O}}_{2\text{peak}}$, MET_peak_, and WR_peak_ increase after the training period in IG. These findings are in agreement with previous studies even if a different methodology of exercise prescription was applied due to the different characteristics of patients (obese and elderly). For instance, it is well known that when exercise is prescribed referring to absolute parameters (velocity or watt) corresponding to AerT_ge_, exercise capacity can improve in obese adult T2DM [15, 38]. In addition, Morton et al. showed that walking at a heart rate of 80% HR_peak_, type 2 diabetic subjects improved peak and submaximal cardiorespiratory responses. However, the AerT_ge_ of our subjects was lower than the patients evaluated in other studies (80% HR_peak_). Our positive results support the hypothesis that the lower exercise intensity was balanced by the longer training period used in our protocol.

Moreover the workload corresponding to AerT_ge_ improved after training. In support of this, also Larose et al. [46] observed that workload at AerT_ge_ increased while $\%{\dot{\text{V}}\text{O}}_{2\text{peak}}$ at AerT_ge_ remained unchanged after 6 months of walking and cycling exercise at 60–75% HR_max⁡_ for 25–45 min, three times a week in adult type 2 diabetic subjects.

In conclusion, a 3-month aerobic exercise training based on HR, corresponding to subjects' AerT_ge_, improved maximal exercise capacity and had positive effects on glycated haemoglobin levels. Thus, the prescription of exercise intensity PA, according to AerT_ge_, should become more frequent in obese diabetic populations, since training at this intensity improves aerobic capacity, cost effectiveness of treatment and could also increase adherence to physical activity in obese subjects.

## Figures and Tables

**Table 1:** Anthropometric measures, lipid profile, and glycated haemoglobin for the intervention group (IG) and the control group (CG) at baseline and after 3-month period.

	IG (*n* = 15)		CG (*n* = 15)	
	T0	T3	T0	T3
Weight (kg)	87.6 ± 19.5	85.0 ± 17.8^*^	87.0 ± 22.6	87.0 ± 22.1
BMI (kg/m^2^)	33.6 ± 7.7	32.6 ± 7.1^*^	33.0 ± 5.4	32.9 ± 5.3
Fat mass (%)	31.5 ± 10.4	29.5 ± 9.5^*^	32.5 ± 4.2	31.1 ± 7.1
Abdominal circumference (cm)	117.8 ± 17.5	115.6 ± 15.9^*^	107.0 ± 12.2	105.4 ± 12.8
Glycated haemoglobin (mmol/mol)	49.9 ± 8.3	45.3 ± 7.3^*^	53.0 ± 12.2	45.0 ± 5.6^*^
Total cholesterol (mg/dL)	210.6 ± 57.9	217.1 ± 56.9	163.8 ± 33.1	162.0 ± 37.6
HDLC (mg/dL)	46.6 ± 16.2	48.7 ± 13.2	43.8 ± 12.9	46.7 ± 13.0
LDLC (mg/dL)	122.9 ± 51.6	136.3 ± 40.1	96.0 ± 51.2	99.3 ± 22.6

BMI: body mass index; HDLC: high density lipoprotein cholesterol; LDLC: low density lipoprotein cholesterol.

^∗^ *P* < 0.05 versus T0.

**Table 2:** Physiological parameters for the intervention group (IG) and the control group (CG) at baseline and after 3-month period.

	IG (*n* = 15)		CG (*n* = 15)	
	T0	T3	T0	T3
$\dot{\text{V}}$O_2peak_ (ml·kg^−1^ min^−1^)	15.9 ± 3.0	18.5 ± 3.2^*^	18.6 ± 4.2	17.9 ± 5.7
MET_peak_	4.5 ± 0.8	5.3 ± 1^*^	5.3 ± 1.2	5.1 ± 1.6
WR_peak_ (W)	65.7 ± 25.9	78.6 ± 26.1^*^	96.0 ± 23.0	88.3 ± 20.0
%HR_max_ (%)	77.1 ± 9.5	77.9 ± 7.9	79.0 ± 5.8	80.3 ± 7.5
%$\dot{\text{V}}$O_2peak_ at AerT_ge_ (%)	57.7 ± 11.9	51.7 ± 11.6	58.1 ± 12.1	59.9 ± 16.5
%HR_max_ at AerT_ge_ (%)	57.7 ± 8.3	56.1 ± 8.0	60.0 ± 7.6	60.0 ± 6.7
%HRR at AerT_ge_ (%)	24.4 ± 12.2	21.6 ± 9.8	18.9 ± 4.7	18.4 ± 7.2
WR at AerT_ge_ (w)	20.4 ± 4.7	28.0 ± 8.2^*^	20.8 ± 9.5	23.3 ± 0.3
ΔHR (bpm)	21.5 ± 11.6	18.9 ± 9.5	14.2 ± 3.9	14.3 ± 7.7
MET at AerT_ge_	2.6 ± 0.6	2.6 ± 0.6	3.1 ± 0.6	2.9 ± 0.6

$\dot{\text{V}}$O_2peak_: peak oxygen uptake; MET: metabolic equivalent; WR_peak_: peak work rate.

HR_max_: maximum heart rate; HRR: heart rate reserve; ΔHR: heart rate at AerT_ge_-heart rate at rest.

^∗^ *P* < 0.05 versus T0.
